# Properties of Antibiotic-Resistant Bacteria Isolated from Onsite Wastewater Treatment Plant in Relation to Biofilm Formation

**DOI:** 10.1007/s00284-017-1428-2

**Published:** 2018-01-20

**Authors:** Łukasz Jałowiecki, Joanna Żur, Joanna Chojniak, Helene Ejhed, Grażyna Płaza

**Affiliations:** 1 Department of Environmental Microbiology, Institute for Ecology of Industrial Areas, Katowice, Poland; 2 Department of Biochemistry, Faculty of Biology and Environmental Protection, University of Silesia in Katowice, Katowice, Poland; 3 Natural Resources & Environmental Effects, IVL Swedish Environmental Research Institute, Stockholm, Sweden

## Abstract

The aim of the present study was to determine some properties of antibiotic-resistant bacterial strains isolated from onsite wastewater technology in relation to biofilm formation, e.g., autoaggregation and motility. Additionally, biosurfactant production by the isolates was also evaluated. The ability of selected strains to develop a biofilm was assessed by using the crystal violet method, which allows to indirectly quantify the attached bacterial biomass (live, dead cells, and polysaccharides as well). Obtained results showed that 19 of the analyzed strains were able to produce biofilm after 72 h of incubation. The low values of surface tension in the range between 28 and 36 mN/m were observed in the bacteria, which are not able to produce biofilm or be classified as weak biofilm producers. Among biofilm-forming strains the highest autoaggregation index was observed for *Mycobacterium brumae* and *Bacillus alcalophilus*. Noteworthy, that some strains capable of biofilm formation showed no aggregation abilities or were characterized by low autoaggregative properties. The results of visual autoaggregation assay showed no visible flocs after given time of incubation. The results from motility test demonstrated that most of the analyzed strains were motile. Noteworthy, that up to now literature data about physiology, biofilm formation, and autoaggregative capabilities of bacteria isolated from onsite wastewater technology are very limited and this paper gives the information on the antibiotic-resistant bacteria with ability to form biofilm. Thus, the present study points to develop novel bioinocula in antibiotic degradation and to reach novel biofilm-dispersing agents produced by various bacteria that can be used as disinfectants or surface-coating agents to prevent microbial surface colonization and biofilm development.

## Introduction

Biofilm is a complex, densely packed multicellular community of microorganisms attached to a surface or interface enclosed in a self-produced polymeric matrix consisting mainly of polysaccharides, proteins, lipids, water, extracellular DNA, and humic substances [17–19]. Microorganisms in biofilms are characterized by physiological and genetic differentiation and increased tolerance to xenobiotics and toxic compounds. Varied gene expression and different metabolic degradation pathways exhibited by bacteria in biofilms allow biodegradation, transformation, immobilization, or detoxification a wide range of pollutants, e.g., antibiotics, heavy metals, petroleum, or pesticides [9, 21]. Moreover, the biofilm matrix, particularly the EPS (extracellular polymeric substances) compounds, provides protection against harsh environmental conditions, antimicrobial agents, shear forces, acidification, predation, or UV damage [28]. Simultaneously, microorganisms in biofilms have limited access to oxygen and nutrients mainly due to the mass transfer limitation, which results in the formation of altered microenvironments within the biofilm matrix.

Biofilms are the source of persistent infections of many pathogenic microbes. They are responsible for dental caries and nosocomial infections, as well as a variety of other infections and diseases. Industrially, biofilms are also harmful in many cases, for instance: natural biofilms can reduce heat transfer in heat exchangers and cooling towers, decompose reverse osmosis membranes, corrode metal surfaces, and contaminate food processing equipment [25]. With the cells embedded in a polysaccharide matrix, biofilms are highly resistant to antibiotics and have higher genetic transformation frequencies than planktonic cells. However, there are several successful examples of the positive use of biofilms that are called beneficial biofilms [19].

Aggregation of bacteria is considered as one of the essential steps in biofilm formation [22, 27]. There are two types of bacterial aggregation, auto- and coaggregation. The first of them is defined as the specific adhesion and recognition between genetically identical bacteria, while the second type refers to genetically distinct microorganisms [15]. For the most bacterial species, surface factors (flagella, lipopolysaccharides), extracellular polymeric substances, quorum-sensing signals, or environmental signal molecules are crucial factors involved in autoaggregation and biofilm formation processes [4]. Simoes et al. [27] noticed that aggregation depends on a wide range of interactions, such as synergism, antagonism, mutualism, competition, or commensalism, occurring between bacteria. Motility of bacteria determined by the presence of fimbriae, pili, or flagella may facilitate colonization and moving of bacteria across the different surfaces. In some bacterial species, flagella may promote attachment to abiotic and biotic surfaces and recruitment of motile cells from the planktonic phase of bacteria. However, the involvement of flagella in biofilm formation depends on several different factors, e.g., bacterial strain, surface, stage of biofilm, or culture conditions [10]. The formation of biofilm is one of the significant means for survival of microorganisms in their surrounding environment. Those microbes, which form biofilm around them, are comparatively more resistant to antimicrobial agents. When the microbes are in the planktonic form they are comparably less tolerant to this antibiotic.

The aim of this study was to evaluate the properties of antibiotic-resistant bacteria isolated from onsite wastewater technology in relation to biofilm formation, e.g., autoaggregation, motility, and biosurfactant production in order to select the isolates for creation of the bacterial consortia with antibiotics degradation abilities.

## Materials and Methods

### Bacterial Strains

The bacterial strains were isolated from the tested field at PIA (Development and Assessment Institute in Waste Water Technology, RWTH Aachen Germany) from the fluidized bed bioreactor as the example of onsite wastewater treatment plants. It operates on a principle of a fluidized bed biological reactor with fluidized media providing a high active surface for microorganisms growing on it. In the bioreactor, most of the microorganisms are immobilized on the small, fluidized units of carrier media such as black plastic pieces, which make the treatment process to be operated with a minimal biomass wash-out. Suspended microorganisms in the bioreactor, which are released from the fluidized media, are also present. Microorganisms, which are also sloughed from the surface of carrier media, were collected as part of a liquid sample.

The biochemical profiles of the bacteria evaluated by EcoPlates microplates, GEN III Omnilog® ID System and phenotypic microarray (PM11 and PM12) were performed in the previous study [5, 14].

### Antibiotic-resistance Detection

Antibiotic resistance of bacteria was carried out against 30 antibiotics by the standard disc diffusion method. The strains were grown in Luria–Bertani (LB) medium containing (g L^−1^): casein peptone (10.0) yeast extract (5.0) and NaCl (5.0) at 30 °C for 24 h. Then, the bacterial suspensions were adjusted to OD_600nm_ 0.5 (ca. 10^7^–10^8^ CFU x dm^−3^), and 100 µl was spread onto Mueller–Hinton agar plates (Oxoid). The antibiotic-impregnated discs (Oxoid) were put on these freshly prepared bacterial lawns and incubated at 30 °C for 24 and 48 h. The degree of resistance or sensitivity of the strains was determined by the measurements of lightened zones (expressed in mm) around the disc and by comparing with the standard antibiotic disc sensitivity testing method (EUCAST 2011). The strains which showed resistance or intermediate were classified as “resistant.” All others were classified as sensitive. The antibiotics used in this test are presented in Table 1.

**Table 1:** List of antibiotics used in this study

Active substance	Symbol	Level (µg)	Group	Effects
Amikacin	AK	30	Aminoglycosides	Protein synthesis disruption/binding to the 30S-subunit of ribosome
Amoxicillin	AML	30	Aminopenicillins	Binding to penicillin-binding protein 1A (PBP-1A) inside the bacterial cell well
Ampicillin	AMP	25	Aminopenicillins	Inhibition of bacterial cell wall synthesis/interferes with autolysin inhibitor
Azithromycin	AZM	15	macrolides	Protein synthesis inhibition/binding to the 50S ribosomal subunit of the bacterial 70S ribosome
Aztreonam	ATM	30	Beta-lactams/ monobactams	Inhibition of bacterial cell wall synthesis/high affinity for penicillin-binding protein 3 (PBP3)
Cefaclor	CEC	30	Cephalosporins	Binding to penicillin-binding proteins (PBPs) inside the bacterial cell wall
Cefadroxil	CFR	30	Cephalosporins	Binding to specific penicillin-binding proteins (PBPs) inside the bacterial cell wall
Cefoxitin	FOX	30	Cephalosporins	Inhibition of cell wall synthesis
Ceftaroline	CPT	5	Cephalosporins	Inhibition of cell wall synthesis
Ceftazidime	CAZ	30	Cephalosporins	Binding to specific penicillin-binding proteins (PBPs) inside the bacterial cell wall
Ciprofloxacin	CIP	10	Chemotherapeutic	Inhibition of the topoisomerase II (gyrase) and topoisomerase IV, required for DNA replication, recombination, transcription, and strand supercoiling repair
Doripenem	DOR	10	Carbapenems	Inhibition of penicillin-binding proteins (PBPs), mostly 1a, 1b, 2, 3
Doxycycline	DO	30	Tetracycline	Reversibly binds to the 30 S ribosomal subunits, blocking the binding of aminoacyl-tRNA to the mRNA/ inhibiting bacterial protein synthesis
Ertapenem	ETP	10	Carbapenems	Binding to penicillin-binding proteins (PBPs)
Erythromycin	E	30	Macrolides	Reversibly binding to the 50S subunit of bacterial ribosomes
Gentamicin	CN	120	Aminoglycosides	Bind to 30S-subunit proteins and 16S rRNA
Imipenem	IPM	10	Carbapenems	Inhibition of cell wall synthesis/binding to penicillin-binding proteins (PBPs)
Metronidazole	MTZ	50	Chemotherapeutic	Prodrug/disruption of DNA helical structure/inhibiting bacterial nucleic acid synthesis and resulting in bacterial cell death
Minocycline	MH	30	Tetracycline	Binding to the 30S ribosomal subunit/preventing the binding of tRNA to the mRNA-ribosome/interfering with protein synthesis
Mupirocin	MUP	200	Mupirocin	Inhibition of bacterial protein and RNA synthesis/reversibly binds to bacterial isoleucyl-tRNA synthetase
Nalidixic acid	NA	30	Chemotherapeutic	Binding reversibly to DNA/interfering with synthesis of RNA and proteins
Neomycin	N	10	Aminoglycosides	Binding to specific 30S subunit proteins and 16S rRNA
Netilmicin	NET	30	Aminoglycosides	Irreversibly bind to specific 30S subunit proteins and 16S rRNA
Nitrofurantoin	F	300	Chemotherapeutic	Activated by nitrofuran reductase/inhibition of DNA, RNA, protein, and cell wall synthesis
Norfloxacin	NOR	10	Chemotherapeutic	Inhibition of the enzymes topoisomerase II (DNA gyrase) and topoisomerase IV
Novobiocin	NV	30	Aminoglycosides	Aminocoumarin/inhibition of bacterial DNA gyrase/competitive inhibitors of the ATPase reaction catalyzed by GyrB
Ofloxacin	OFX	5	Chemotherapeutic	Acts on DNA gyrase and topoisomerase IV/inhibiting cell division
Piperacillin	PRL	100	Penicillin	Binding to specific penicillin-binding proteins (PBPs) inside the bacterial cell wall
Rifampicin	RD	30	Rifampicin	Inhibition of DNA-dependent RNA polymerase/suppression of RNA synthesis and cell death
Teicoplanin	TEC	30	Peptide	Inhibition of peptidoglycan polymerization, resulting in inhibition of bacterial cell wall synthesis and cell death
Ticarcillin	TIC	75	Penicillin	Able to prevent cross-linking of peptidoglycan during cell wall synthesis
Tobramycin	TOB	30	Aminoglycosides	Binding irreversibly to one of two aminoglycoside-binding sites on the 30S ribosomal subunit/inhibiting bacterial protein synthesis
Trimethoprim	W	5	Chemotherapeutic	Binding to dihydrofolate reductase/inhibition of the reduction of dihydrofolic acid (DHF) to tetrahydrofolic acid (THF)
Trimethoprim- sulfamethoxazole	SXT	25	Chemotherapeutic	Inhibition of the enzymatic conversion of pteridine and p-aminobenzoic acid (PABA) to dihydropteroic acid by competing with PABA for binding to dihydrofolate synthetase
Vancomycin	VA	30	Peptide	Inhibition of cell wall biosynthesis

### Biofilm Formation Using the Microtiter Plates

The biofilm assay was performed according to the procedure described by Stepanović et al. [32]. Each well of 96-well tissue culture plate (TPP® Tissue Culture Plate 96F) was filled with 180 μl sterile LB medium and 20 μl of individual overnight culture strain dilution of 1:100 in fresh LB medium and incubated for 24, 48, and 72 h at 28 °C. After the incubation period, the wells were washed three times with 200 μl/well sterile phosphate-buffered saline (PBS, pH 7.3), emptied, and left to dry. Afterwards, the plates were fixed with 200 μl/well methanol (Sigma-Aldrich) for 30 min, dried, and then stained with 200 μl/well of 0.1% crystal violet water solution (CV) (Sigma-Aldrich) for 20 min. After staining followed by brief drying, 200 μl/well of 96% ethanol (Sigma-Aldrich) was added into each well in order to resolubilize the dye bound to the adherent cells and incubate for 30 min. Negative controls were obtained by incubating the wells only with 200 μl/well LB medium, without bacteria. The optical density (OD) of the obtained solution was measured at 600 nm (OD_600_) using a microtiter plate reader (Plate Reader AF2200, Eppendorf). Examined strains were divided into the following categories using the classification of Stepanović et al. [32]: non-biofilm producer (referred as 0; OD less than or equal to ODc); weak biofilm producer (1; OD greater than ODc and less than or equal to 2xODc); moderate biofilm producer (2; OD greater than 2xODc and less than or equal to 4xODc); and strong biofilm producer (3; OD greater than 4xODc). This classification was based upon the cut-off value called ODc [ODc means average OD of negative control + (3 x standard deviations of OD negative control)] which allows for separate biofilm producing and non-biofilm producing strains.

### Autoaggregation Assay

Visual autoaggregation behavior of the 30 isolates was performed according to Simoes et al. [27] and Cisar et al. [6] with certain modifications. Bacteria were cultivated for 24 h at 28 °C in LB medium with the following composition g L^−1^: 10 tryptone (Sigma-Aldrich), 10 NaCl (Sigma-Aldrich), and 5 yeast extract (BioMérieux). The cells were harvested at stationary phase by centrifugation at 5000*×g* for 15 min, washed twice, and resuspended in phosphate-buffered saline (PBS, pH 7.3) and adjusted to OD at a wavelength of 600 nm to approximately 1.0 (10^8^ CFU ml^−1^). The bacterial suspensions (2.5 ml) were mixed by vortexing by 10 s, then transferred onto 24-well plate (Falcon), and allowed to settle. Degree of autoaggregation was determined after 0, 2, and 24 h of incubation at 28 °C. After the incubation period, a 0.2 ml of upper portion of the suspensions was transferred onto 96-well plate and OD_600_ was measured (Plate Reader AF2200, Eppendorf). The autoaggregation percentage was expressed as (1 − A_t_)/A_0_ × 100, where A_t_ means the absorbance at time *t* = 2 and 24 h and A_0_ the absorbance at time *t* = 0. All experiments were performed in triplicate.

### Motility Test

Motility test was performed according to Atkinson et al. [1] with minor modifications. Bacteria were cultivated for 24 h at 28 °C in LB medium. 2 μl of each strain culture was dropped in the middle of plates with semisolid motility test medium containing 1% tryptone (Sigma-Aldrich), 0.5% NaCl (Sigma-Aldrich), and 0.3% agar (Oxoid) and incubated for 3 days at 28 °C. The motility halos were measured at 24, 48, and 72 h. All experiments were performed in triplicate.

### Surface Tension (ST) Measurements

To study the surface activity (biosurfactant production) of the strains, supernatant samples of the centrifuged cultures were measured for ST using a Du Nöuy ring with a tensiometer SIGMA 702 (Attension). ST measurements were carried out at room temperature after dipping a platinum ring in the solution for enough time to attain equilibrium conditions. To calibrate the instrument, the ST of pure water was measured. Measurements were repeated at least three times, and an average value was used to express the surface activity of each sample. Attension software was used to analyze all data.

### Statistical Analyses

Pearson correlation (*P* = 0.05) between biofilm formation (absorbance) and autoaggregation (percentages) for all times of incubation was performed in Statistica 10.0.

## Results and Discussion

Resistance of the examined bacterial strains to various antibiotics is presented in Table 2. The obtained results showed that about 20–40% of the analyzed strains were resistant to 14 antibiotics, while < 20% of the strains were resistant to 20 antibiotics. The bacterial strains were also analyzed for multiple antibiotic resistance (MAR). Among the tested strains, *Paenibacillus azoreducens*, resistant to 35 of the investigated antibiotics (all tested antibiotics) belong to eight various chemical classes. *Pseudomonas fragi, Stenotrophomonas rhizophila*, and *Sphingobacterium multivorum* were resistant to 22, 21, and 17 antibiotics, respectively. About 20% of the tested bacteria showed a 2–5 MAR (i.e., resistance from 2 to 5 antibiotics of the 37 antibiotics tested), while 47% were resistant to more than 5 antibiotics. Detailed description of the antibiotic susceptibility of bacteria isolated from onsite WWT facilities is presented by Jałowiecki et al. [13].

**Table 2:** Resistance of the bacteria to selected antibiotics

Species	Antibiotics	
	Number	Symbol
*Streptococcus australis*	4	ATM,CEC,CAZ,MTZ
*Pseudomonas fluorescens*	2	NA,MTZ
*Stenotrophomonas maltophilia*	3	DOR,ETP,MTZ
*Pseudomonas fragi*	21	AML,AMP,ATM,CEC,CFR,FOX,CPT,CAZ,DOR,ETP,IPM,MTZ,MUP,NA,N,F,NV,PRL,RD,TEC,W
*Stenotrophomonas rhizophila*	20	AK,AML,AMP,CEC,CFR,FOX,CPT,CAZ,DOR,ETP,IPM,MTZ,MUP,N,F,NV,PRL,RD,TEC,SXT
*Microbacterium flavescens*	9	AMP,ATM,CPT,CAZ,MTZ,MUP,RD,W,SXT
*Lactobacillus coryniformis subsp. coryniformis*	2	MTZ,TIC
*Microbacterium maritypicum*	8	AMP,ATM,CPT,CAZ,MTZ,MUP,RD,W
*Alcaligenes faecalis ss faecalis*	7	ATM,CAZ,MTZ,PRL,RD,TEC,W
*CDC group II-E A*	9	CAZ,MTZ,F,NV,PRL,TEC,TIC,W,VA
*Pseudomonas chlororaphis subsp. aurantiaca*	1	E
*CDC group II-H*	1	MTZ
*Flavobacterium hydatis* (26 C)	6	ATM,CFR,CAZ,MTZ,W,SXT
*Flavobacterium resinovorum*	1	MTZ
*Mycobacterium brumae*	1	MTZ
*Flavobacterium hydatis*	1	MTZ
*Bacillus horti*	2	PRL,TEC
*Variovorax paradoxus*	1	MTZ
*Bacillus alcalophilus*	1	MTZ
*Acinetobacter johnsonii*	1	MTZ
*Chryseobacterium balustinum*	10	AML,AMP,ATM,CPT,CAZ,MTZ,MUP,RD,W,SXT
*Aeromonas bestiarum*	10	AMP,CFR,DOR,ETP,IPM,MTZ,NA,NV,PRL,TEC
*Enterococcus haemoperoxidus*	2	MTZ,NA
*Paenibacillus azoreducens*	35	AK,AML,AMP,AZM,ATM,CEC,CFR,FOX,CPT,CAZ,CIP,DOR,DO,ETP,E,CN,IPM,MTZ,MH,MUP,NA,N,NET,F,NOR,NV,OFX,PRL,RD,TEC,TIC,TOB,W,SXT,VA
*Carnobacterium divergens*	4	MTZ,TEC,W,VA
*Streptococcus criceti*	7	AMP,CAZ,,MTZ,F,TEC,W,VA
*Pseudomonas fulva*	4	MTZ,TEC,W,VA
*Flavobacterium tirrenicum*	1	MTZ
*Sphingobacterium multivorum*	17	AK,AML,AMP,ATM,CPT,E,IPM,MTZ,MUP,N,F,PRL,TEC,TOB,W,SXT,VA
*Serratia marcescens subsp. marcescens*	11	AML,AMP,CEC,CFR,DO,MTZ,F,NV,RD,TEC,VA

**Fig. 1:**
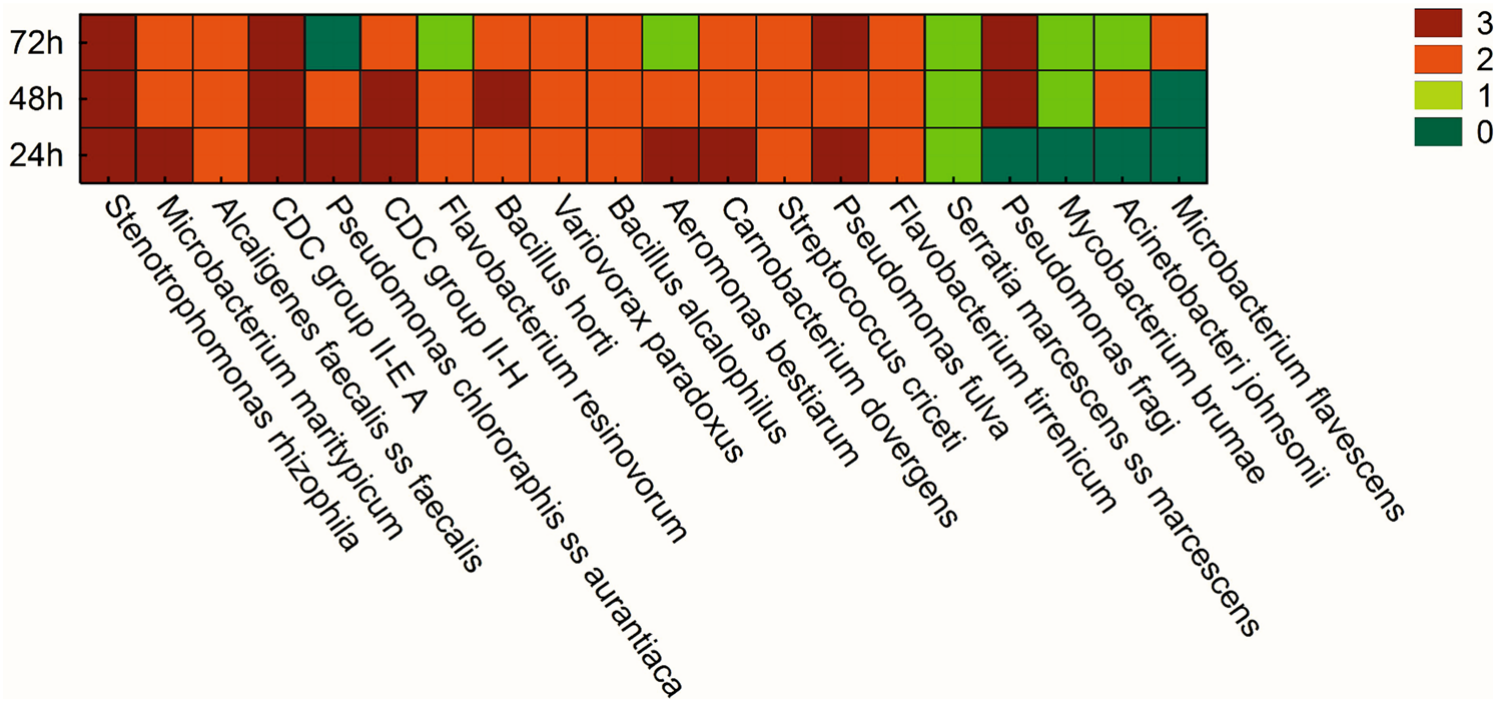
Classification of biofilm producer bacteria according to Stepanović et al. [8, 15] 0 - nonbiofilm producer; 1 - weak biofilm producer; 2 - moderate biofilm producer; 3 - strong biofilm producer

Apart from the evaluation of antibiotic susceptibility profile of bacteria, the ability of selected strains to develop a biofilm on polystyrene microtiter dishes was assessed by using the CV method. This method allows to indirectly quantify the attached bacterial biomass (live and dead cells, polysaccharides). Obtained results showed that most of the analyzed strains (19) from total 30 were able to form biofilm after 72 h of incubation (16/30 after 24 h; Table 3; Fig. 1). Interestingly, *Pseudomonas chlororaphis ss aurantiaca* strain was able to produce biofilm only after 24 h of incubation. For example, *Pseudomonas fulva, Mycobacterium maritypicum, Aeromonas bestiarum, Carnobacterium divergens*, and *Stenotrophomonas rhizophila* strains were classified as strong biofilm producers. *Streptococcus australis, Pseudomonas fluorescens, Stenotrophomonas maltophilia, Paenibacillus azoreducens*, and *Enterococcus haemoperoxidus,* for example, were unable to form biofilm even after 72 h of incubation. In Table 3, the relation between biofilm formation and surface activities measured by surface tension is presented. The low values of surface tension in range between 28 and 36 mN/m were observed in the bacterial strains which were not able to produce biofilm or be classified as weak biofilm producers, for example, *S. australis, P. fluorescens, S. maltophilia, P. chlororaphis ss aurantiaca, CDC group II-H, Flavobacterium resinovorum, Mycobacterium brumae, Bacillus horti, Acinetobacter johnsonii, Enterococcus haemoperoxidus, Serratia marcescens ss marcescens*.

**Table 3:** Relation between biofilm formation and surface activity (Mean ± Stand. Dev.)

Species	Properties			
	Biofilm (600 nm)			Surface tension (mN/m)
	24 h	48 h	72 h	72 h
*Streptococcus australis*	0.00	0.06 ± 0.05	0.10 ± 0.03	36.35 ± 0.08
*Pseudomonas fluorescens*	0.00	0.09 ± 0.001	0.00	32.96 ± 0.04
*Stenotrophomonas maltophilia*	0.00	0.00	0.00	32.15 ± 0.12
Pseudomonas fragi*	0.00	1.36 ± 0.13	1.27 ± 0.02	56.77 ± 0.10
Stenotrophomonas rhizophila*	1.76 ± 0.09	1.23 ± 0.13	0.89 ± 0.06	63.97 ± 0.16
Microbacterium flavescens*	0.00	0.00	0.73 ± 0.08	51.14 ± 0.15
*Lactobacillus coryniformis subsp. coryniformis*	0.00	0.00	0.00	64,62 ± 0,2
Microbacterium maritypicum*	0.81 ± 0.12	0.56 ± 0.1	0.45 ± 0.1	60.43 ± 0.03
Alcaligenes faecalis subsp. faecalis*	0.56 ± 0.13	0.55 ± 0.1	0.40 ± 0.08	51.25 ± 0.08
CDC group II-E A*	2.75 ± 0.2	3.77 ± 0.15	2.19 ± 0.12	63.21 ± 0.43
Pseudomonas chlororaphis subsp. aurantiaca*	0.76 ± 0.1	0.65 ± 0.02	0.00	32.33 ± 0.05
CDC group II-H*	0.98 ± 0.11	0.80 ± 0.05	0.43 ± 0.05	28.89 ± 0.26
*Flavobacterium hydatis* (26 C)	0.00	0.00	0.00	52.84 ± 0.02
Flavobacterium resinovorum*	0.44 ± 0.09	0.53 ± 0.1	0.36 ± 0.07	28.69 ± 0.13
Mycobacterium brumae*	0.18 ± 0.01	0.19 ± 0.02	0.21 ± 0.03	28.41 ± 0.01
*Flavobacterium hydatis*	0.17 ± 0.05	0.07 ± 0.001	0.00	48.95 ± 0.03
Bacillus horti*	0.66 ± 0.1	0.86 ± 0.12	0.60 ± 0.01	34.27 ± 0.29
Variovorax paradoxus*	0.45 ± 0.18	0.70 ± 0.03	0.59 ± 0.08	57.06 ± 0.03
Bacillus alcalophilus*	0.62 ± 0.11	0.47 ± 0.07	0.51 ± 0.04	56.71 ± 0.12
Acinetobacter johnsonii*	0.0	0.27 ± 0.04	0.30 ± 0.07	29.92 ± 0.02
*Chryseobacterium balustinum*	0.00	0.00	0.00	43.11 ± 0.01
Aeromonas bestiarum*	1.04 ± 0.11	0.60 ± 0.1	0.20 ± 0.01	61.79 ± 0.39
*Enterococcus haemoperoxidus*	0.00	0.00	0.00	32.78 ± 0.04
*Paenibacillus azoreducens*	0.01 ± 0.001	0.00	0.00	54.67 ± 0.12
Carnobacterium divergens*	0.81 ± 0.14	0.55 ± 0.12	0.47 ± 0.09	51.23 ± 0.36
Streptococcus criceti*	0.72 ± 0.06	0.44 ± 0.07	0.60 ± 0.04	57.22 ± 0.08
Pseudomonas fulva*	0.85 ± 0.07	0.43 ± 0.09	0.75 ± 0.04	55.83 ± 0.07
Flavobacterium tirrenicum*	0.59 ± 0.01	0.54 ± 0.01	0.45 ± 0.03	42.50 ± 0.04
*Sphingobacterium multivorum*	0.00	0.00	0.00	52.63 ± 0.06
Serratia marcescens subsp. marcescens* Control (LB medium) Water	0.37 ± 0.11	0.21 ± 0.01	0.20 ± 0.01	30.97 ± 0.12 68.64 ± 0.81 70.13 ± 0.23

*Biofilm-forming strains

The dispersal properties of biosurfactants have been shown to rival those of conventional inhibitory agents against bacterial and yeast biofilms. This makes them suitable candidates for use in new generations of microbial dispersal agents and for use as adjuvants for existing microbial suppression or eradication strategies [2]. New insights into biofilm physiology have now enabled researchers to design more effective bacterial inhibition/ dispersal strategies. There are two main inhibitory strategies, based on the formulation of new antibiofilm compounds and the construction of biofilm-resistant surfaces [34]. Some of the most promising candidates for the inhibition of bacterial biofilms have come from biological surface-active agents (biosurfactants), since they are characterized by strong anti-adhesive, antimicrobial, and biofilm disruption properties [2, 20]. Many authors demonstrated the abilities of biosurfactants to disperse a biofilm of pathogenic microbial species by decreasing the cells viability and reducing the bacterial adhesion properties. The possible mechanism of such actions is related to binding of the biosurfactants molecules to cell wall components or its surface, which results in severe changes in outer membrane hydrophobicity. The insertion of surfactants into bilayer structure of cell membrane may result in disruption of its integrity. As a response to the increasing concentration of fengycin, the reorganization of membrane lipids into bilayer discs composed of fengycin micelles and lipids from membrane has been reported. The negative influence of biosurfactants on both Gram-negative and Gram-positive strains is related to the releasing of LPS molecules from the outer membrane or to the formation of transmembrane pores, which results in increased permeability of the cell wall, respectively [23, 30]. Satputea et al. [25] discuss the various roles of biosurfactants molecules in association with biofilm formation.

Literature data about biofilm formation capabilities of onsite wastewater technology microorganisms are very scarce, although application of biofilms in removal, bioremediation or biotransformation of organic pollutants, heavy metals, pharmaceutical, or personal care products (PPCPs) is well documented [9]. Microorganisms in biofilms are characterized by higher tolerance towards harsh environmental conditions compared with their free-floating counterparts. Van Houdt and Michiels [33] noticed that biofilm formation process depends on several factors such as the bacterial surface, the surface for attachment, and surrounding medium. In recent years, bacterial biofilms have been widely utilized to degrade, neutralize, and mineralize various contaminations in wastewater-activated sludge or as recently in onsite wastewater technology. Microorganisms in communities are able to persist in different metabolic states, which increase efficiency of xenobiotics degradation. Moreover, multicellular and porous structure of multispecies biofilm allow for nutrients transport or accumulation of metabolites from the environment. Bacteria in sessile mode of growth are also able to communicate through quorum sensing (QS) and to exchange genetic material. As Edwards and Kjellerup [9] point out in the case of several PPCPs removal, e.g., non-steroidal anti-inflammatory drug diclofenac, lipid regulator gemfibrozil, and chemotherapeutic agent trimethoprim, membrane biofilm reactor was more efficient than traditional activated sludge. It is widely known that occurrence and fate of antibiotics in the environment may lead to the selection of antibiotic-resistant bacteria (ARB) [24, 26]. As many authors highlight, WWTPs often constitute the main source of pharmaceuticals released into the environmental matrices. Conn et al. [7] reported about the presence of 30 different organic wastewater contaminants (OWCs) including endocrine-disrupting compounds, antimicrobial agents, heavy metals, or disinfectants. On the other hand, Stanford et al. [31] reported about excellent removal of nonylphenols, estrone (E1), 17β-estradiol (E2), estriol (E3), and 17α-ethinylestradiol (EE2) from five onsite wastewater systems with aerobic and anaerobic sand filters. Besides this, development of more effective technologies, which will prevent the propagation of antibiotic-resistant bacteria and antibiotic-resistance genes, is still necessary. A promising alternative for removal of antibiotics are bioremediation methods with the use of bacterial strains able to degrade xenobiotically. Simultaneously, many authors suggest that immobilization of bacterial consortia on both organic and inorganic carriers increases degradation capabilities and provides protection against harsh environmental conditions, e.g., acidification, heavy metals, or high osmotic pressure. One of the crucial factors for immobilization of bacteria is biofilm formation ability. Characterization and determination of several bacterial features like cell wall properties or autoaggregation are also valuable for effective whole cell immobilization and degradation studies.

**Fig. 2:**
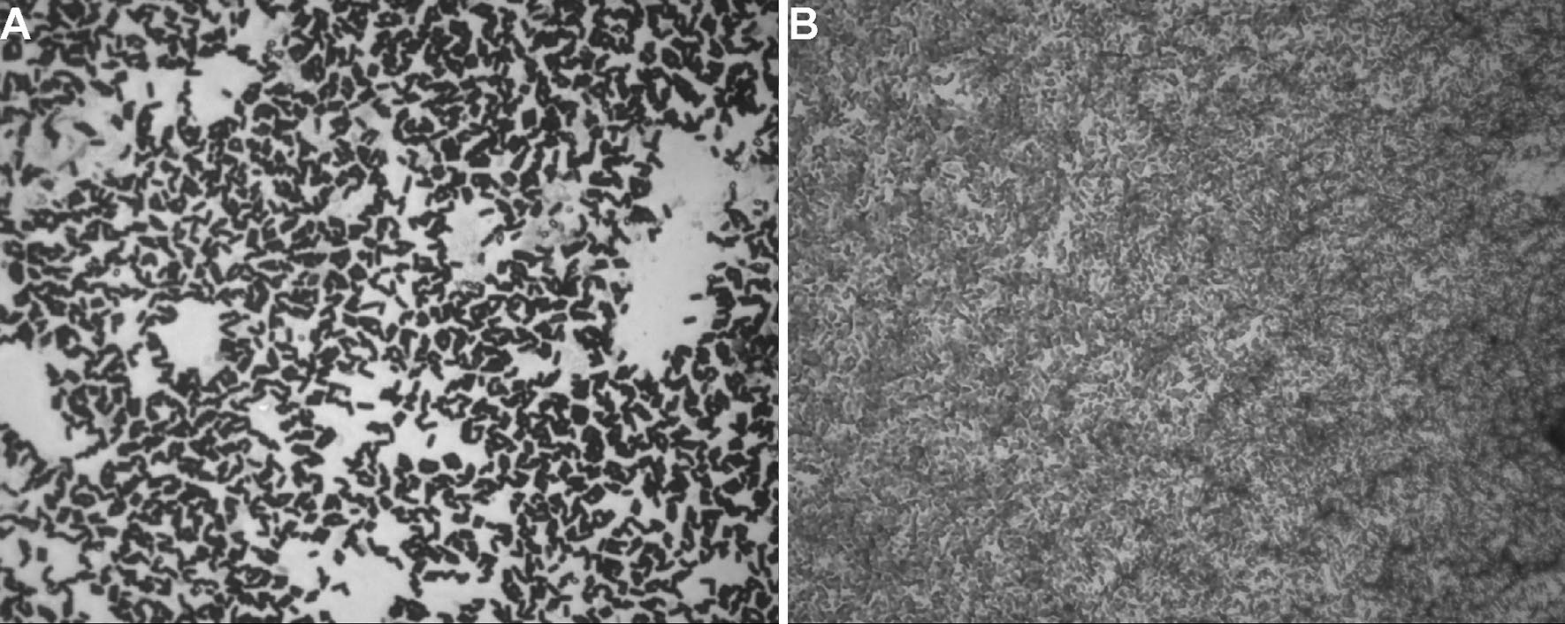
Light microscope image of autoaggregation after 24 h of incubation in PBS buffer (pH 7.3). **A** *Microbacterium maritypicum* **B** *Aeromonas bestiarum*. Magnification x500

All examined strains exhibited varied autoaggregation index increasing with the time of incubation (Table 4). Among biofilm-forming strains, the highest autoaggregation index was observed for *M. brumae* and *Bacillus alcalophilus*. Noteworthy, that some strains are able to form biofilm, e.g., *A. johnsonii, Streptococcus criceti*, and *P. fulva* showed, no aggregation abilities were characterized by low autoaggregative properties, e.g., *S. rhizophila, B. horti, B. alcalophilus, Variovorax paradoxus, P. fulva, C. divergens*, or *Flavobacterium tirrenicum*. Strong autoaggregating phenotype was observed also for *Microbacterium flavescens, Microbacterium maritypicum* (Fig. 2a), *CDC group II-H,* and *A. bestiarum* (Fig. 2b) strains. Beside this, the results of visual autoaggregation assay showed no visible flocs after given time of incubation. Simoes et al. [27] reported that some species of bacteria are not able to form flocs without the presence of other bacteria species. Aggregation is also considered as one of the essential steps in communication among microorganisms and ecological interactions, e.g., adaptation and succession, which lead to colonization and subsequent biofilm formation [35]. In natural settings, multispecies biofilm is primarily a mode of bacterial growth, where auto- and coaggregation processes mediate the formation of multicellular matrix and juxtapositioning of bacteria near to taxonomically favorable species present within the biofilm. In recent years, the role of initial attachment and bacterial aggregation in biofilm formation has been increasingly highlighted, since autoaggregation and coaggregation capabilities of bacteria facilitated the attachment to inert and biotic surfaces. Involvement of bacterial surface components, particularly EPSs, LPSs, outer membrane proteins (OMPs), and flagella, in combination with microbial signals in autoaggregation and biofilm formation processes has been widely documented [29]. Moreover, the absence of biofilm-associated structures indicates that cell hydrophobicity and auto- or coaggregation properties are crucial factors responsible for bacterial adherence [3]. A positive correlation between autoaggregation and biofilm formation abilities was demonstrated, e.g., for *Sinorhizobium meliloti* strains [29] isolated from root nodules of alfalfa plants and *Myroides odoratus* isolated from fish *Oreochromis mossambicus* [12]. Moreover, obtained results indicated that both phenomena were dependent on the same adhesive forces. Similar observations were made by Kos et al. [16] which observed the strong relationship between adhesion and aggregation ability of probiotic *Lactobacillus acidophilus* M92 strains. On the other hand, Basson et al. [3] observed no correlation between autoaggregation and biofilm formation for 29 *Flavobacterium johnsoniae*-like isolates. In this study, weak correlations were observed between biofilm formations after 24, 48, and 72 h of incubation and 2 h of autoaggregation (*P* = 0.05; *r* = 0.13; *r* = 0.21; *r* = 0.09), respectively. Similarly, weak or negative correlations were observed also between 24, 48, and 72 h of incubations and 24 h of autoaggregation (*P* = 0.05; *r* = 0.02; *r* = − 0.033; *r* = − 0.74), respectively. Aggregation of bacteria is one of the essential processes, which plays an important role in both biofilm formation and various ecological interactions. Microorganisms in biofilms are characterized by physiological and structural heterogeneity and diverse gene expression. Increased tolerance of biofilms to various environmental pollutions or toxic compounds and their ability to immobilize ensure higher degradation and accumulation capacity of biofilms compared with planktonic cells. Motility of bacteria, dependent on the presence of flagella or pili, is one of the crucial factors which mediate the adherence of bacteria to different surfaces and hence biofilm formation.

**Table 4:** Autoaggregation ability after 2 and 24 h incubation at 28 °C in PBS (pH 7.3)

Strains	Autoaggregation (%)	
	2 h	24 h
*Streptococcus australis*	14.82	17.25
*Pseudomonas fluorescens*	16.63	76.69
*Stenotrophomonas maltophilia*	0	0.63
*Pseudomonas fragi* ^a^	16.5	45.21
*Stenotrophomonas rhizophila* ^a^	9.06	67.24
*Microbacterium flavescens* ^a^	0.61	87.46
*Lactobacillus coryniformis subsp. coryniformis*	8.08	49.87
*Microbacterium maritypicum* ^a^	0	91.51
*Alcaligenes faecalis subsp. faecalis* ^a^	10.89	40.23
*CDC group II-E A* ^a^	17.19	36.23
*Pseudomonas chlororaphis subsp. aurantiaca* ^a^	33.95	53.25
*CDC group II-H* ^a^	17.19	81.40
*Flavobacterium hydatis* (26 C)	19.61	91.23
*Flavobacterium resinovorum* ^a^	0.16	4.26
*Mycobacterium brumae* ^a^	35.19	87.63
*Flavobacterium hydatis*	0	0
*Bacillus horti* ^a^	19.15	24.99
*Variovorax paradoxus* ^a^	31.61	51.01
*Bacillus alcalophilus* ^a^	38.44	81.31
*Acinetobacter johnsonii* ^a^	0	2.03
*Chryseobacterium balustinum*	7.21	94.12
*Aeromonas bestiarum* ^a^	1.28	91.71
*Enterococcus haemoperoxidus*	2.48	5.07
*Paenibacillus azoreducens*	7.38	79.25
*Carnobacterium divergens* ^a^	4.89	39.36
*Streptococcus criceti* ^a^	0	0
*Pseudomonas fulva* ^a^	0	0
*Flavobacterium tirrenicum* ^a^	0.69	42.61
*Sphingobacterium multivorum*	0.91	53.38
*Serratia marcescens subsp. marcescens* ^a^	9.57	24.68

^a^Biofilm-forming strains

**Fig. 3:**
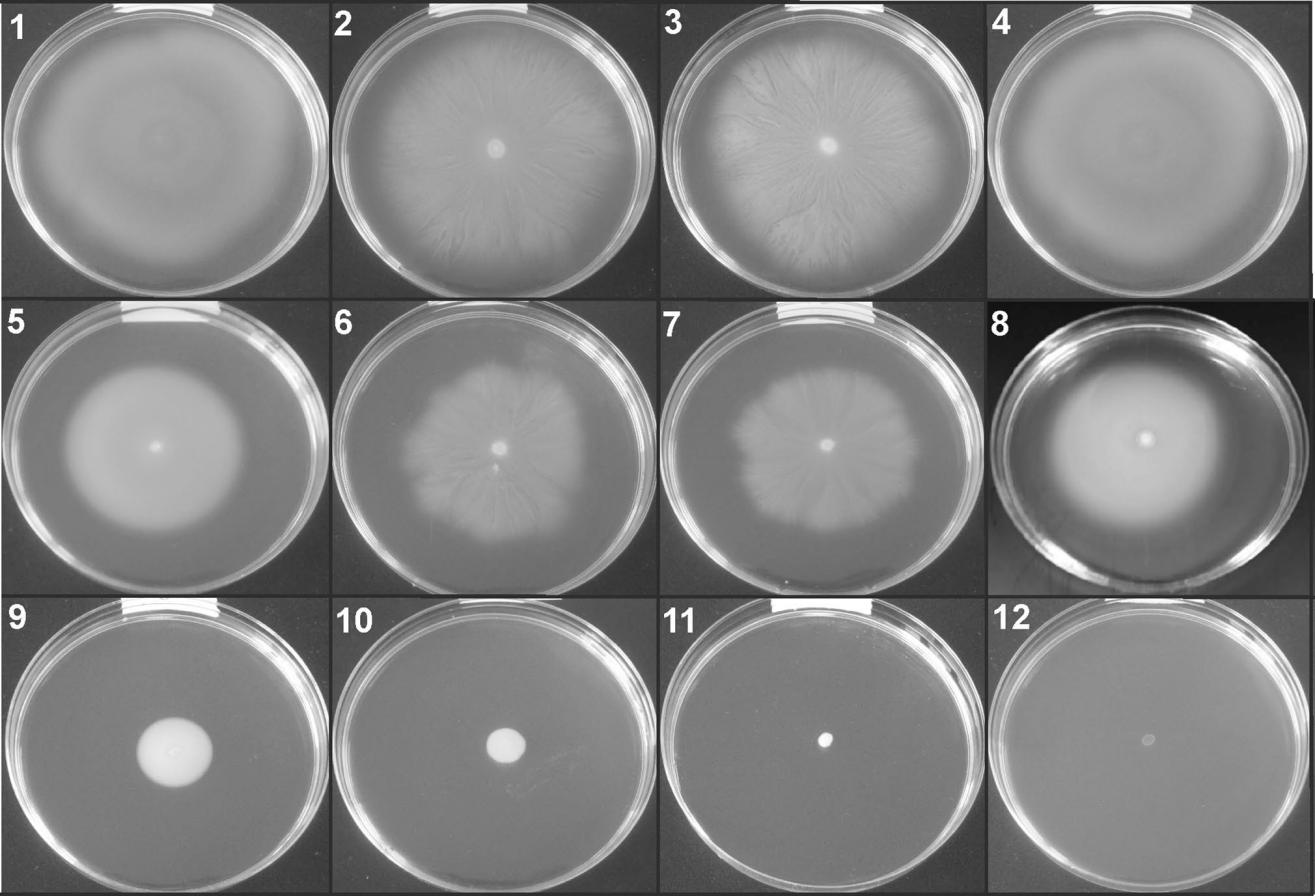
Motility of selected bacteria on 0.3% agar swim plates after 72 h of incubation. 1. Bacillus horti (diameter of motility halo: 53 mm); 2. Bacillus alcalophilus (diameter of motility halo: 49 mm); 3. Stenotrophomonas maltophilia (diameter of motility halo: 48 mm) 4. CDC group II-H (diameter of motility halos: 44 mm) 5. Variovorax paradoxus (diameter of motility halo: 36 mm) 6. Flavobacterium resinovorum (diameter of motility halo: 38 mm) 7. Pseudomonas fulva (diameter of motility halo: 37 mm) 8. Lactobacillus coryniformis ss coryniformis (diameter of motility halo: 13 mm) 9. Stenotrophomonas rhizophilia (diameter of motility halo: 9 mm) 10. Flavobacterium hydatis (diameter of motility halo: 7 mm) 11. Sphingobacterium multivorum (nonmotile) 12. Paenibacillus azoreducens (nonmotile). Diameters of motility halos were the same after 24 h, 48 h and 72 h of incubation

The results from motility test demonstrated that most of the analyzed strains (27/30), except *Chryseobacterium balustinum, Paenibacillus azoreducens*, and *Sphingobacterium multivorum*, were motile. In Fig. 3, 12 bacterial strains with different motility properties are presented (Fig. 3). Surface-associated structures such as pili and flagella are considered as important factors involved in bacterial motility. Lack of biofilm-forming capabilities in non-motile bacterial mutants is well documented [3]. Our results also confirmed those findings; all three non-motile bacterial strains simultaneously were not able to from biofilm. Flagellar motility is one of the essential factors for initial contact between the bacterial cell and surface and biofilm formation capabilities under static culture conditions for several bacterial strains, e.g., *Escherichia coli, Listeria monocytogenes*, and *Yersinia enterocolitica* [1, 11]. Van Houdt and Michiels [11] reported that flagella may influence the attachment and biofilm formation via several different mechanisms due to the involvement in reaching the surfaces, facilitating growth and spread of a maturing biofilm, and finally flagella act as cell wall appendages mediating directly the attachment of bacteria to biotic and abiotic surfaces.

## Conclusions

Results obtained in this study widely illustrate a great variability in biofilm formation and autoaggregation abilities exhibited by antibiotic-resistant bacteria isolated from onsite wastewater technology. Particular capabilities of examined strains will be helpful in the construction of bacterial consortia able to degrade antibiotics and their subsequent immobilization on different carriers. Future analysis will be focused on specific interactions between species, e.g., coaggregation and biofilm formation in multispecies systems.

Future studies addressing the role of antibiotic-resistant bacteria with biofilm formation will be of great interest, due to the development of novel bioinocula in antibiotic degradation. Also, the preliminary screening of biosurfactant-producing bacteria with the biofilm properties is the new direction in developing novel biofilm-dispersing agents that can be used as disinfectants or surface-coating agents to prevent detrimental microbial surface colonization and biofilm development.
